# Microglial pathology

**DOI:** 10.1186/s40478-014-0142-6

**Published:** 2014-09-26

**Authors:** Wolfgang J Streit, Qing-Shan Xue, Jasmin Tischer, Ingo Bechmann

**Affiliations:** Department of Neuroscience, University of Florida College of Medicine and McKnight Brain Institute, PO Box 100244, 32610-0244 Gainesville, FL USA; Institute of Anatomy, University of Leipzig, Leipzig, Germany

**Keywords:** Senescence, Oxidative damage, Chronic neuroinflammation, Glial dysfunction, Neurodegeneration

## Abstract

This paper summarizes pathological changes that affect microglial cells in the human brain during aging and in aging-related neurodegenerative diseases, primarily Alzheimer’s disease (AD). It also provides examples of microglial changes that have been observed in laboratory animals during aging and in some experimentally induced lesions and disease models. Dissimilarities and similarities between humans and rodents are discussed in an attempt to generate a current understanding of microglial pathology and its significance during aging and in the pathogenesis of Alzheimer dementia (AD). The identification of dystrophic (senescent) microglia has created an ostensible conflict with prior work claiming a role for activated microglia and neuroinflammation during normal aging and in AD, and this has raised a basic question: does the brain’s immune system become hyperactive (inflamed) or does it become weakened (senescent) in elderly and demented people, and what is the impact on neuronal function and cognition? Here we strive to reconcile these seemingly contradictory notions by arguing that both low-grade neuroinflammation and microglial senescence are the result of aging-associated free radical injury. Both processes are damaging for microglia as they synergistically exhaust this essential cell population to the point where the brain’s immune system is effete and unable to support neuronal function.

## Introduction

The idea of “diseased microglia” represents a recent conceptual development in neuropathology that was first raised in the context of Creutzfeldt-Jakob disease [1]. *Diseased* microglia are different from *activated* microglia, in that they are incapacitated cells whereas activated microglia are cells capably responding to injury. Microglial disability and dysfunction have now captured the imagination of neuroscientists made apparent by a number of excellent recent reviews with different perspectives on the subject [2–6]. Following the conceptual introduction of diseased microglia by v. Eitzen et al. [1], additional clues about the existence of unhealthy microglia came from immunohistochemical studies describing abnormal morphological features of microglial cells in the aged human brain [7], termed microglial dystrophy and thought to reflect microglial senescent degeneration. The microglial dysfunction hypothesis of Alzheimer’s disease (AD) claiming that neurofibrillary degeneration is the result of weakening microglial support was published in the same year [8]. Microglial morphology has been and continues to be of interest to neuroscientists due to its ever-changing nature and the initial morphological assessments suggesting pathological dysfunction have been strengthened by recent genetic discoveries, which are discussed in the above mentioned reviews. It seems a milestone has been reached in terms of a renewed appreciation of the importance of diminutive microglial cells whose main function in normal brain is to make sure neurons are well protected and connected [9–12]. As the idea of microglial dysfunction gains momentum and its implications for improved understanding of dementia pathogenesis are being realized, we consider it important to summarize the current state of the art from a neuropathological perspective, as well as to discuss potential causes and consequences of microglial degeneration.

In this paper, we focus on microglial cell morphology and phenotype as seen by immunohistochemical staining of brain sections. Morphological plasticity is a characteristic feature of microglia that is evident even in the static images of traditional microscopy, as shown here. Modern microscopy methods allowing live cell imaging have confirmed what has long been suspected from cell culture studies [13], namely that microglia are on the move all the time [14,15]. In light of this restlessness it is probably time to abandon the term “resting microglia”, although it may still serve the purpose of providing an antonym to “activated microglia”. It remains a challenge to translate morphological plasticity into more detailed functional plasticity and to further investigate the meaning of altered morphological appearances, such as hypertrophy and dystrophy. While it is known, in general, that hypertrophy is associated with activation, i.e. increased metabolic and phagocytic activity in energized microglia, and that dystrophy is associated with senescent degeneration in burned-out microglia, there are many stages in between these two extremes as both activation and senescence are progressive processes with many gradations [3,16]. Microglial activation ensues rapidly following most perturbations of CNS homeostasis, and the intensity and extent to which it occurs is commensurate with the severity of the disturbance. For example, minimally activated microglia are also known as primed microglia and they have been described mostly in experimental mouse model systems [17–19], and to a lesser extent in human brain [20]. In contrast, maximally activated microglia appear as rounded brain macrophages that are also referred to as amoeboid microglia [21,22]. Senescent changes in microglia may develop gradually over decades during human aging and likely occur as a consequence of multiple influences on the CNS microenvironment, notably free radical-mediated oxidative damage. However, microglial pathology can also occur as a result of sudden extreme stress, as suggested by findings from experimental animals. A good approach towards understanding functional changes that occur in microglia as they undergo morphological transformations in human brain is through characterization of their immunophenotype, which can be quite heterogeneous due to up- and down-regulation of multiple proteins and receptors [23,24]. Microglia harbor a large assortment of intracellular and surface antigens and there exists an arsenal of antibodies directed against various microglial antigens, some of which can be linked to specific functions [22]. As the number of microglia-binding antibodies continues to grow, it is likely that these reagents will facilitate an ever more refined *in situ* assessment of microglial phenotypes which in conjunction with morphological changes will provide information about cell function and dysfunction in a variety of disease states. I*n situ* description and evaluation of microglia in human brain in terms of their morphology and phenotype is essential for elucidating the role these cells play in neurodegenerative disease pathogenesis. Human histopathology is also important for establishing relevance and represents the first line of investigation for neurodegenerative conditions. Together with human genetic studies, as well as experimental transgenic or other animal or cell culture models of disease addressing molecular mechanisms, true insights into disease pathogenesis can be gained.

### Microglial activation – is it pathological?

The currently widespread opinion that activated microglia can be both beneficial and detrimental leaves open the possibility that microglial activation is harmful under certain conditions, and destructive microglial neurotoxicity as a possible factor in neurodegenerative disease has been discussed on numerous prior occasions (for reviews see [5,25–27]). However, both resting and activated microglia perform beneficial neuron-supporting functions and they exert harmful effects only if they become senescent, diseased, or die unable to perform their normal supportive roles. Microglial activation has been described as a natural consequence to CNS injury in countless, acute experimental situation since the days of del Rio-Hortega [28] revealing how the brain responds to injury and engages in wound healing. The purportedly detrimental effects exerted by activated microglia in the context of chronic neuroinflammation are inferred from studies showing neurotoxic effects of rodent microglia *in vitro* under artificial conditions. Neither the time scales nor the microenvironment created *in vitro* are representative of human brain [29], and while simplified *in vitro* systems may show what microglia are theoretically capable of doing when stimulated to the maximal extent possible, these results are not validated by human neuropathology and they cannot address the complexities of neurodegenerative disease pathogenesis with direct relevance. A major discrepancy between microglial activation *in vitro* and *in vivo* is that *in vitro* experiments use the proverbial sledgehammer method (LPS or similar) to achieve maximal microglial stimulation *in vitro,* producing maximal and readily measurable effects, while at the same time it is recognized that the intensity of microglial activation in the normally aged and AD brain is consistent with only minimal, low-level neuroinflammation [30–32]. To equate such low-intensity microglial activation in human brain with artificially produced high-intensity microglial neurotoxicity *in vitro* constitutes a flaw in scientific reasoning explained possibly by immature judgment or premature ambition.

### Microglial pathology in the aged brain

It is currently thought that chronic, low-level inflammation is associated with major degenerative diseases of aging including those affecting the CNS [30,33]. Histopathological studies suggesting that microglial activation occurs with normal brain aging have contributed towards this notion by showing increased microglial expression of interleukin-1α and histocompatibility antigens (HLA-DR) in humans [16,34–36], seemingly consistent with observations in experimental animals showing increased expression of major histocompatibility complex (MHC) class II antigens and upregulation of interleukin-1 on acutely activated microglia and macrophages in a variety of brain lesions [37–42]. Animal studies have also demonstrated increased expression of MHC class II antigens on microglia with aging, as well as morphological and phenotypic transition to a more macrophage-like appearance [43–47]. Together with literature describing fluctuations and increases in pro-inflammatory cytokines during aging [18,48], the notion that chronic low-level neuroinflammation accompanies brain aging is thus firmly established. However, what is missing from this conception is the inciting stimulus, that is, a definitive cause of aging-associated chronic neuroinflammation remains unidentified. This stands in contrast to other aging-related chronic, non-autoimmune inflammatory conditions outside the CNS which usually have an identifiable cause. For example, in osteoarthritis there is loss/breakdown of articular cartilage which results in bone damage, which in turn elicits a chronic inflammatory reaction, underscoring the point that in order for inflammation to occur there must be damage or injury. Thus, if brain aging causes low-level chronic inflammation it is imperative to understand the reasons for it.

**Figure 1:**
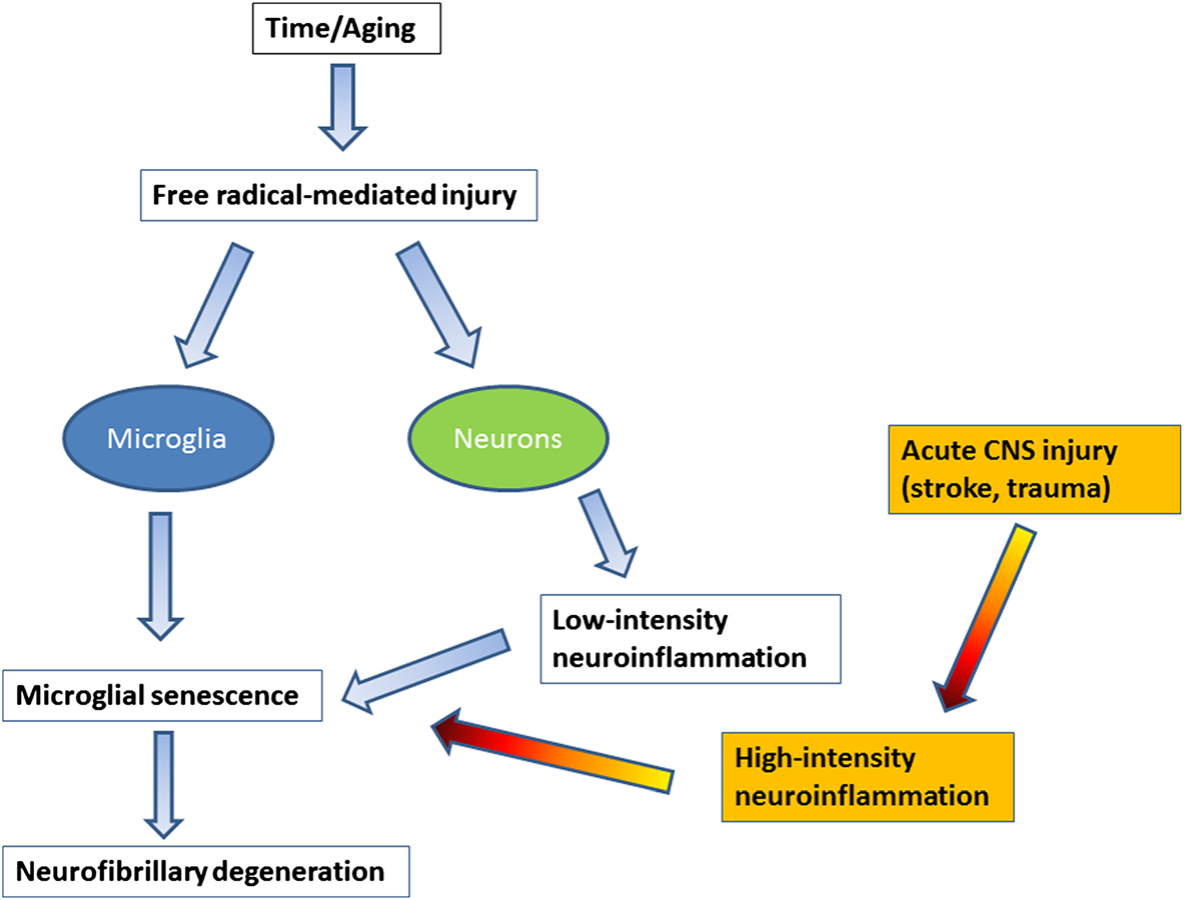
**Microglial senescence may be a key event in aging and Alzheimer’s disease.** Free radical injury occurs naturally during aging affecting both microglia and neurons. It contributes to microglial senescence directly by causing oxidative injury in microglia, and indirectly by causing oxidative injury in neurons which elicits a low-level inflammatory response. The latter causes chronic stress to microglia contributing to exhaustion and senescence. If normal aging is exacerbated by acute injuries eliciting intense inflammation, this may accelerate naturally occurring microglial senescence.

It has been hypothesized that free radical damage in the aging brain causes inflammation [18] but free radical damage in neurons is subtle and non-specific, and therefore difficult to demonstrate histopathologically in normal human brain [49]. Nevertheless, assuming the free radical theory of aging [50] is correct, free radical injury occurs inevitably in aerobic organisms as a consequence of life-long oxygen exposure at all levels producing oxidized nucleic acids and proteins, damaged (senescent) cells and tissues, which is what ultimately accounts for the development of most aging-related diseases. It therefore makes sense that there would be a chronic low-level inflammatory reaction because low-grade inflammation is the predicted tissue response to subtle and gradually developing CNS injury regardless of its cause. Put another way, both chronic low-level inflammation and microglial senescence are indirect and direct results, respectively, of free radical injury associated with aging (Figure 1). Importantly, the mild reactive gliosis (neuroinflammation) that occurs presumably as a result of free radical injury is not an aggressive, neurotoxic type of response and is detrimental only in the sense that it may over time contribute towards development of senescence and dysfunction. The recent demonstration by Raj et al. [17] that DNA damage in a mouse model of genotoxic stress causes mild microglial activation (priming) offers clear support for the idea that free radical injury, which often produces DNA damage, elicits low-level neuroinflammation. In addition, the fact that some measures of aging-related, low-grade neuroinflammation can be attenuated by caloric restriction [47,51] also supports the idea that neuroinflammation is due to free radical damage, since lowered food intake is associated with decreased metabolic activity and concomitantly decreased free radical production [50].

Regarding the microglial changes that occur during aging, these can be seen as being consistent with both mild activation and with senescence. While a rapidly changing immunophenotype and dramatic morphological transformations are part of high-intensity microglial activation after acute CNS lesions, such changes occur only gradually during aging reflecting slowly progressive evolution of a senescent morphology and phenotype, which is similar in its manifestation to low-grade activation described in models of mild CNS injury [52–54]. Microglial secretion of cytokines changes more dramatically during acute, severe injury situation than it does in less severe scenarios [55], and there are aging-associated changes in microglial cytokine production [56–58] which are consistent with the idea that microglia develop a senescence-associated secretory phenotype (SASP). Although thus far the notion of SASP has been discussed in terms of non-CNS cell senescence and tumor suppression rather than in a microglial context [59,60], current understanding of microglial secretory activity during aging and the fact that microglioma is an exceedingly rare tumor [61] are compatible with the SASP concept.

**Figure 2:**
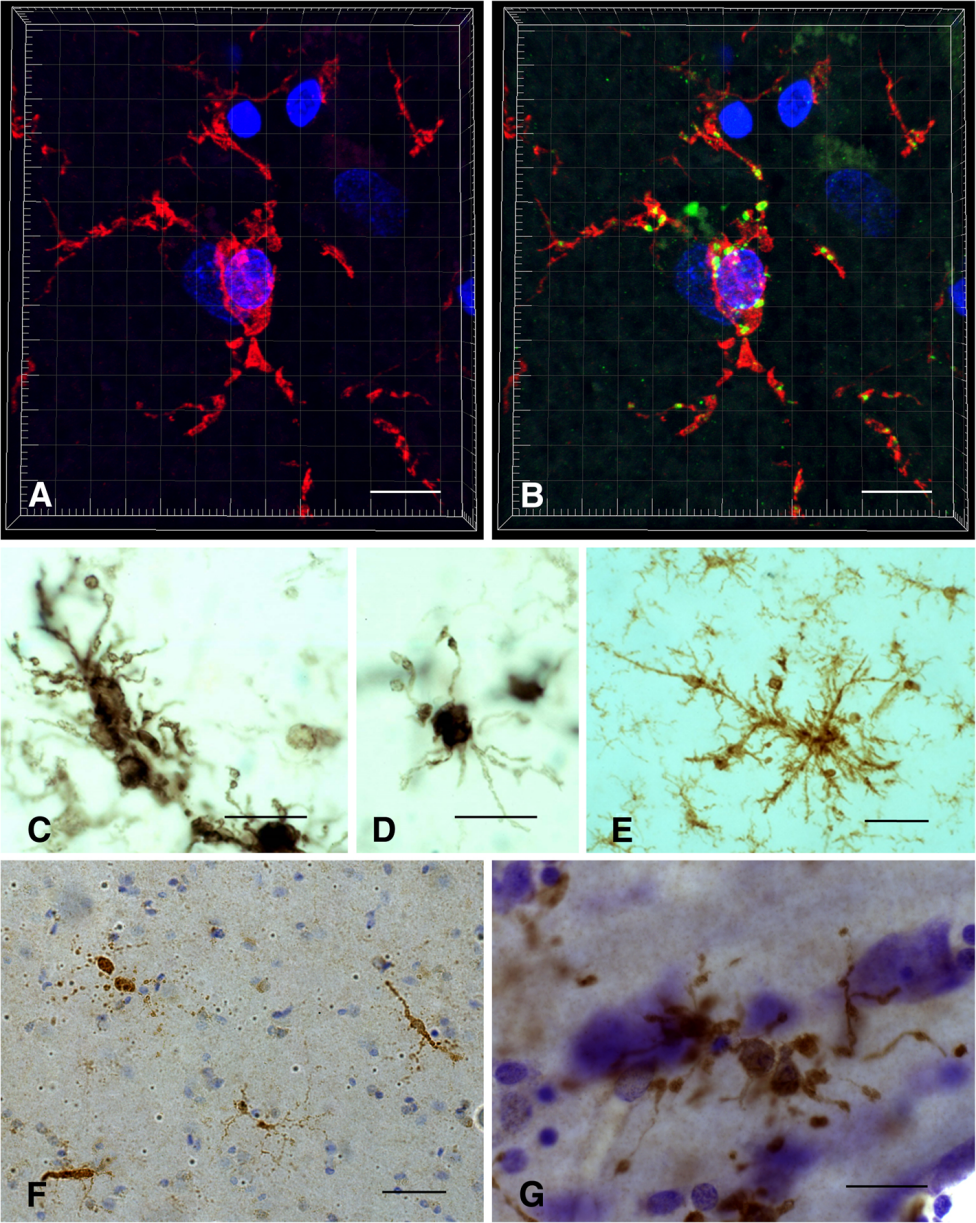
**Dystrophic microglia have many guises and can be stained with different antibodies. A, B** Iba1 (red) and CD68 (green) double immunohistochemical staining of dystrophic microglia in AD brain, as seen by 3-D confocal microscopy. Note fragmentation of cytoplasmic processes and punctate CD68 labeling indicating intracellular location of lysosomal antigen. **C-E**, anti-HLA-DR antigen immunohistochemistry (LN-3 antibody) shows dystrophic spheroid formation **(C)**, atrophic deramified processes **(D)**, and formation of microglial aggregate with spheroids **(E)**. **F**, ferritin-positive fragmented microglia. **G**, Iba1-positive dystrophic microglia with beaded, fragmenting processes and spheroids. All images taken in cerebral cortex of AD subjects. Scale bars: 10 μm **(A, B)**, 20 μm **(C, D, G)**, 40 μm **(E, F)**.

**Figure 3:**
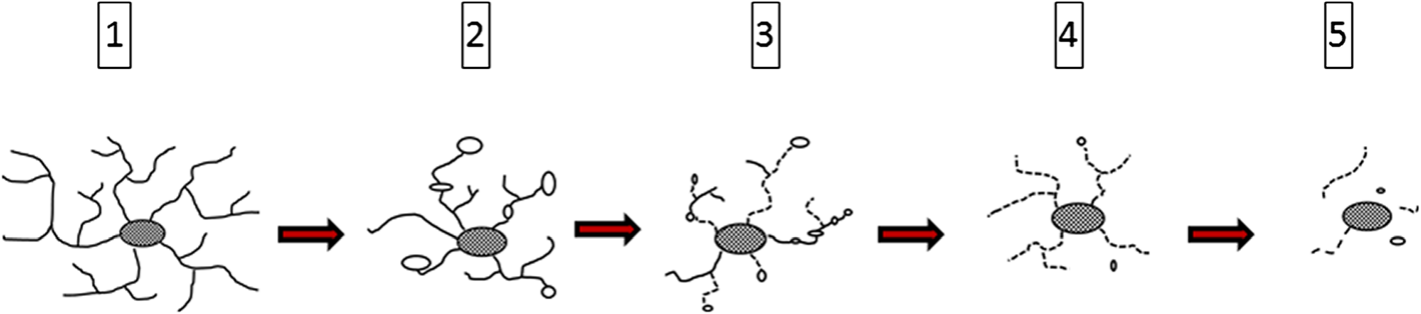
**Schematic representation of cytorrhexis development.** Ramified cells (1) develop spheroidal swellings (2), which progress to beading and partial fragmentation of processes (3), which progresses to complete fragmentation while still maintaining cell contours (4), eventually ending up as scattered fragments (5). Note that the cell nucleus remains intact throughout.

Microglial mitosis, which is part of microglial activation in acute injury situations when there is a sudden demand for more cells to restore homeostasis, is absent or minimal during normal aging when such impromptu demands do not occur. However, when acute CNS injuries happen in aged animals the ability of microglia to divide is unimpaired and even greater than in young animals [62] showing that microglial mitotic potential remains robust in the aged rodent brain. In contrast, replicative senescence (reduced mitotic activity) occurs when there is repetitive CNS injury forcing microglia to undergo repeated bouts of mitosis over the course of one year [63]. Microglial mitosis is difficult to measure reliably during aging in human post-mortem brain, and the most direct evidence for microglial senescence has come from morphological studies. The recognition of microglial dystrophy has provided an indication of microglial senescence in the aged human brain [7]. With aging an increasing proportion of microglial cells display abnormal morphological features, such as shortened, gnarled, beaded, or fragmented cytoplasmic processes, as well as loss of fine ramifications and formation of spheroidal swellings, changes that are designated collectively as microglial dystrophy (Figure 2). Microglial dystrophy reflects the abnormal morphology of senescent microglia because the number of dystrophic microglia increases with aging. In individual cells dystrophy may develop gradually: during early stages microglia develop spheroidal swellings of their processes that can progress to beading and eventually fragmentation leaving pinched–off segments of microglial cytoplasm separated from the perinuclear soma. Depending on how far senescent degeneration has progressed, fragmentation of the microglial cytoplasm (cytorrhexis) may be partial or it may involve the entire cytoplasm leaving multiple fragments that in some cases still delineate the cells’ contours, or in the most advanced case of disintegration are widely scattered about (Figure 3). Interestingly, the microglial cell nucleus undergoes little structural change during this cytoplasmic degeneration and can sometimes be seen in one of the many fragments surrounded by a small rim of perinuclear cytoplasm. Microglial dystrophy may also reveal itself in the form of atrophic, gnarled, or stripped down cytoplasmic processes where fine arborizations have gone missing, similar to what has been observed in senescent neurons [64].

### The difficulty of interpreting microglial morphology and phenotype in human brain

Histopathological studies of microglia can be a valuable diagnostic aid to neuropathologists, a fact that was already recognized many decades ago by del Rio-Hortega, who wrote: “It is sufficient to find the microglial alterations to recognize the areas more deeply affected by the disease” [65]. Histological examination of microglia also provides an opportunity for research because the diversity and heterogeneity of microglial morphology that can be observed in well-stained sections of human brain, particularly with Iba1 antibody, is remarkable and stands in contrast to rodents where microglial morphology is rather uniform. Morphological heterogeneity of microglia in humans can be explained in part by the principle that microglia represent a stable and enduring cell population with low turnover rates, a current notion that has resulted from a rather turbulent series of experiments in mice which occurred in the space of more than two decades: initially the bone marrow chimera studies of Hickey and Kimura [66] claimed a bone-marrow origin of microglia, but subsequent DNA labeling studies suggested stability and persistence of the microglial population [67]. The latter notion was then overturned again by a new set of bone marrow chimera studies inferring substantial replacement of microglia by bone-marrow-derived precursors [68]. However, later experiments demonstrated that irradiation used to create chimeras causes brain damage and hence an influx of the bone-marrow derived precursors [69]. The current view is that microglia derive from primitive yolk-sac macrophages in a Myb-independent manner [70,71]. They are thus genetically different from blood-derived mononuclear cells and their population seems to depend on life-long self-renewal, which renders them susceptible to replicative senescence.

Therefore, this most current notion of microglial stability combined with the longevity of humans, complex human genetics, and the many influences created by diverse human lifestyles, diets, habits of drug consumption (both medicinal and recreational), latent infections, injuries, and systemic diseases, as well as subtle brain pathologies all of which may influence microglial behavior creates a tremendously heterogeneous picture that is reflected in the diverse microglial phenotypes encountered. This morphological heterogeneity of the microglial population can make it difficult to differentiate between non-activated, activated, and senescent (diseased) microglia since some features may not be unequivocal, and thus morphological assessments are best combined with immunophenotypic ones employing several microglial markers to characterize cells.

**Figure 4:**
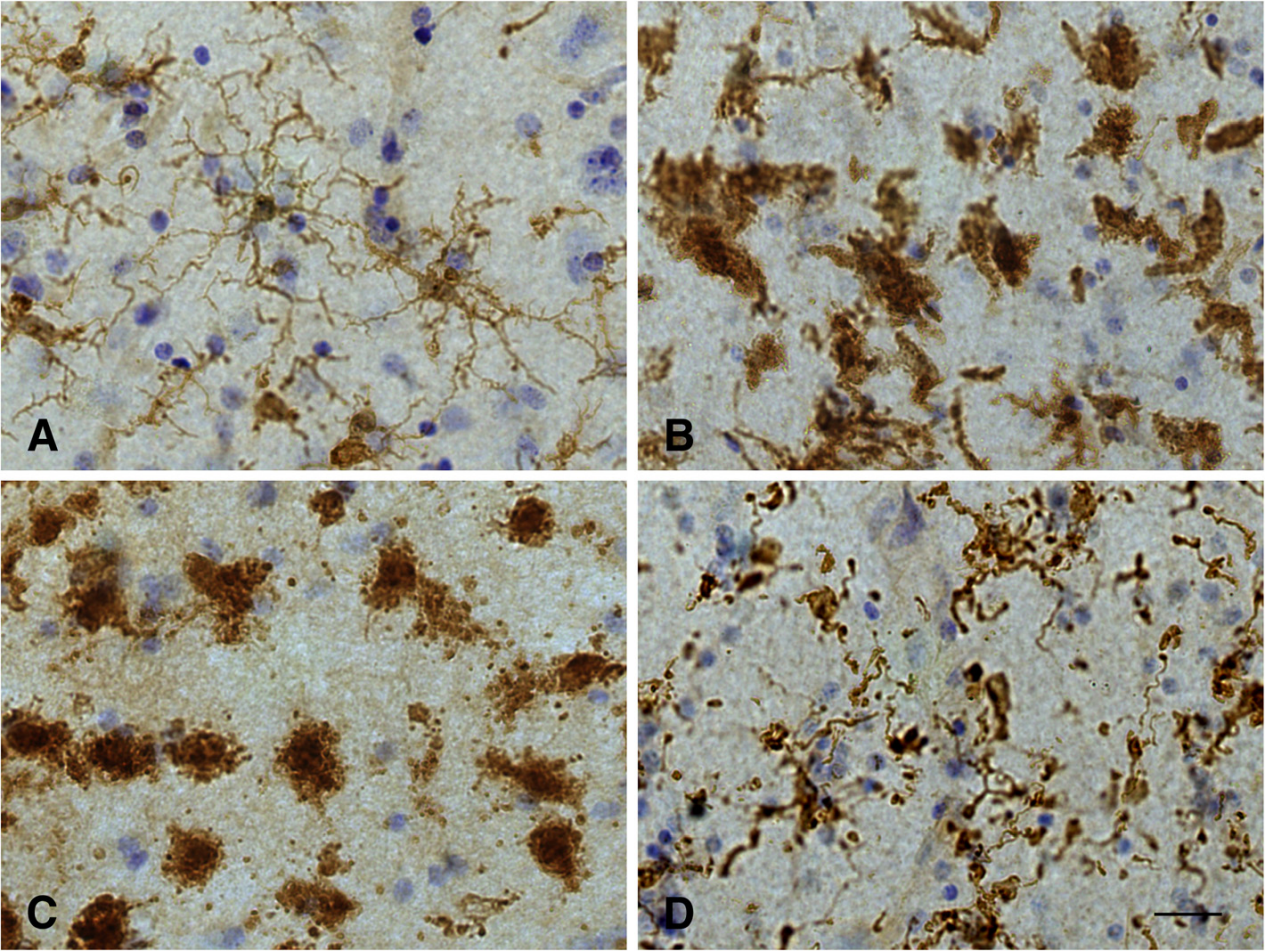
**Different functional states of microglia can be defined morphologically. A**, ramified microglia exhibit highly branched processes with which they explore their surrounding microenvironment. **B**, activated microglia retract processes and become enlarged due to organelle build-up and increased metabolic activity. **C**, phagocytic microglia often appear as rounded brain macrophages. **D**, dystrophic microglia most characteristically display beaded, twisted or fragmented processes. Human cerebral cortex stained with Iba1 antibody. Bar = 20 μm.

A key issue is the reliable identification of and distinction between activated and diseased microglia, because this ultimately provides clues about the cells’ (dys) functional activities in any given region of interest. Since there are no single histochemical markers to facilitate simple differentiation multiple markers together with morphological assessments comprise a conclusive evaluation. Both microglial activation and microglial dystrophy are defined originally by characteristic morphological appearances [7,72]. With the advent of Iba1 antibody [73] a robust reagent has been made available that is resistant to variable tissue fixation and processing protocols and will label all morphological variants of microglia, thereby eliminating largely the problem of inconsistent staining outcomes seen previously with other antibodies under different tissue processing conditions. Iba1 staining allows for excellent, unequivocal morphological differentiation of resting, activated, phagocytic and dystrophic microglia (Figure 4). Staining for HLA-DR antigens is of particular interest since many antibodies directed against these antigens are available and have been used widely to identify activated microglia in human brain, based primarily on the fact that studies in experimental CNS lesions had shown an upregulation of major histocompatibility complex (MHC) antigens by acutely activated microglia [38–40]. However, HLA-DR antigens are present on large numbers of non-activated, ramified microglia in normal human brain [74,75] and HLA-DR expression seems to be affected not only by fixation but by a variety of factors, including gender, age, genotype, and possibly others [34,76]. In some rat strains a high level of constitutive MHC II expression has been reported [77] suggesting that MHC expression could vary also with ethnicity in humans, but this has not been studied. One recent study shows no difference in HLA-DR expression between AD and control subjects [78]. Overall the specific meaning of HLA-DR expression remains unknown and its use for demonstrating activated microglia limited. Its use as a neuroinflammation marker is questionable for reasons already mentioned: widespread expression in normal CNS and high individual variability.

### Senescent microglia in rodents and humans

**Figure 5:**
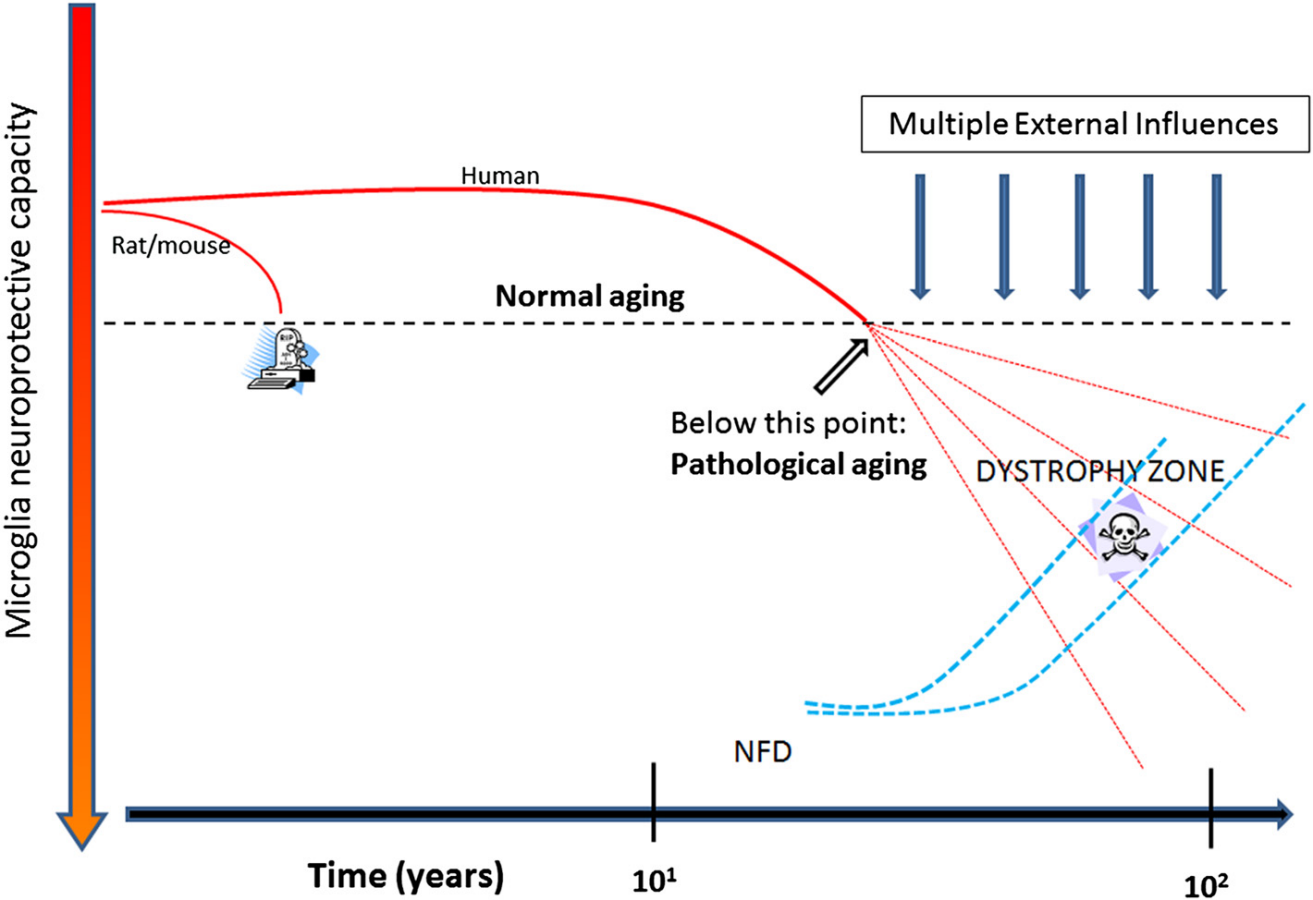
**Histopathological differences in microglial aging between laboratory rodents and humans provide a foundation for normal and pathological aging.** Mice and rats experience only normal aging due to their short life spans and controlled environment, whereas humans who live much longer lives are exposed to diverse environmental influences and lifestyles, including diets, physical and mental activities, drugs, pollutants, comorbidities and infections, all of which may affect the rates at which microglial senescence (dystrophy) occurs, indicated by different trajectories of dashed red lines. As dystrophy occurs to varying degrees there is a decline in microglial neuroprotection and with that neurofibrillary degeneration (NFD) increases at variable rates in different individuals.

Neuroimmunological differences between rodent and human microglia were discussed recently [26], and we would like to not only support the perspectives conveyed by these authors but add to them by pointing out that microglial dystrophy as it appears with aging in human brain is not seen in the aged rodent brain. Dystrophy is part of the aforementioned morphological heterogeneity of human microglia and the fact that it is absent in laboratory rodents speaks to the vast differences in lifespan and environment between rodents and humans. Brain aging happens differently in a short-lived animal raised in a nearly pathogen-free and unchanging environment (incl. a never changing diet) than in a human being where numerous environmental influences can play a role. The one thing common to rodents and men is the air they breathe and the oxygen in it, and it would be safe to say that free radical-mediated oxidative injury plays a role in both rodent and human aging. Microglial cells in rodents do undergo aging-related changes and in light of the constancy of their environment these changes are likely to be caused exclusively by the free radical damage that occurs over time. In the terms of grade-school arithmetic, aging-related changes in rodent microglia represent the least common denominator. Vaughan and Peters provided the first and most comprehensive account of these changes using both light and electron microscopy, and in essence their findings show an increase in cell size due to accumulation of dense inclusions closely resembling lipofuscin deposits [54]. Given the unchanging, sanitary, and uncontaminated lives of laboratory rodents it would be reasonable to assume that occurrence of these lipofuscin deposits is purely a consequence of time passed and is therefore not pathological. Lipofuscin deposits can also be found in human microglia [79], but in addition human cells show those morphological abnormalities that we call microglial dystrophy which may well be a reflection of ongoing pathology. With reference to earlier work [80], one can thus speak of normal and pathological aging also in terms of microglia (Figure 5).

In the same vein, the term neuroinflammation when applied to rodents in the context of normal aging is inappropriate as *inflammation* is inherently tied to injury and pathology, and it would be a stretch (in our minds) to call normal aging a disease. Perhaps more compelling is to point out that these purely aging-related alterations in rodent microglia are not associated with neuronal pathology, unlike in humans where microglial dystrophy and neurofibrillary degeneration appear to be linked.

### Microglial pathology and neurodegeneration

Neurofibrillary degeneration (NFD), or tau pathology, and synapse loss offer the best neuropathological correlates of cognitive impairment and dementia in AD [81–85], and it will be important to elucidate the role that microglial cells play in the development of AD-type neurodegeneration. For quite some time now it has been hypothesized that neurodegenerative changes in AD are caused by pathologically activated microglia thought of as autoaggressive immune effector cells exerting neurotoxic actions. According to the amyloid cascade/neuroinflammation hypothesis, deposits of amyloid-beta (Aβ) protein incite chronic microglial activation and with it a detrimental neuroinflammatory reaction characterized by elevated production of proinflammatory cytokines, neurotoxins, and free radicals that cause neurodegeneration [86,87]. However, the evidence in support of this theory is at best indirect and at worst directly opposed to the idea, and some of the caveats associated with it have already been discussed, i.e. the large discrepancy in the intensity of microglial activation between *in vitro* and *in vivo* studies, the difficulty of reliable identification of activated microglia in human brain, and the fact that neuroinflammation in AD is quite mild and therefore unlikely to be destructive [88]. Additional concerns are raised by findings showing that, a) tau pathology occurs in the absence of Aβ deposits and in the absence of microglial activation [89–91]; b) administration of anti-inflammatory drugs does not slow or reverse tau pathology or cognitive decline [92–94]; c) tau pathology is not initiated or exacerbated by presence of severe neuroinflammation [95]; d) tau pathology does not occur in transgenic animals overexpressing amyloid precursor protein despite extensive Aβ deposition and associated microglial activation [96–98]. For these reasons and others discussed in this paper activated microglia are unlikely suspects in the causation of neuronal pathology associated with AD. Instead it is more likely that degenerating (dystrophic) microglia are linked to NFD because they increase in prevalence as NFD becomes more widespread, they are co-localized with neurofibrillary tangles and senile plaques, and their occurrence precedes the spread of tau pathology [91,99]. The link between microglial and neuronal degeneration is in line also with the fundamental idea of glial-neuronal interdependency, namely, that neuronal well-being is dependent on presence of healthy glial cells. The thought that microglial degeneration is critically important in AD pathogenesis is supported by additional observations showing high incidences of microglial apoptosis in AD brain [100–102].

**Figure 6:**
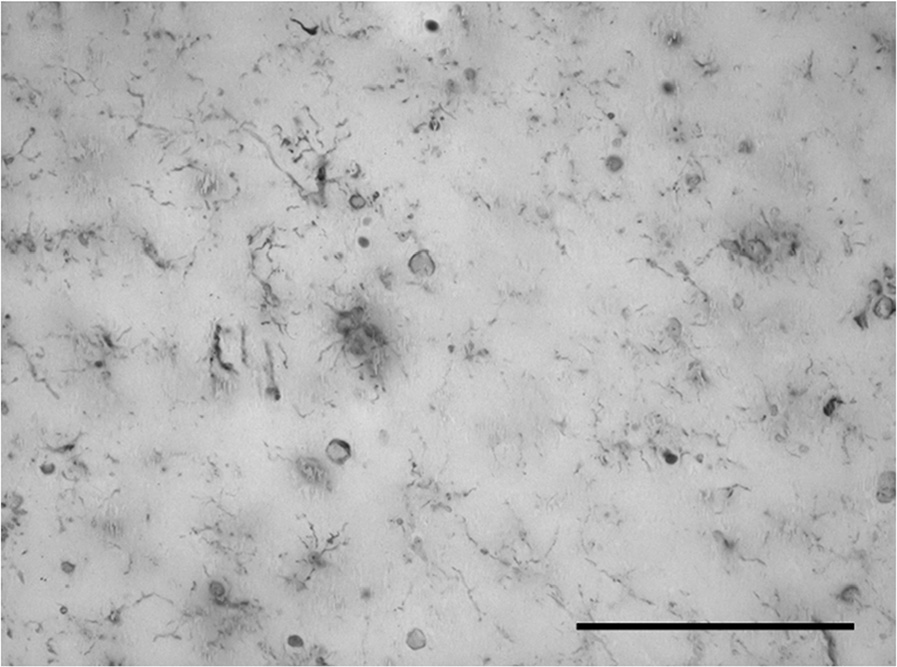
**Widespread dystrophy and absence of brain macrophages are evident in Down syndrome brain.** Iba1-stained section of cerebral cortex from a subject with Down syndrome with Braak stage VI neurodegeneration. Normal microglial morphology is barely discernable in only few cells and the field is mostly occupied by microglial membrane fragments of varying shapes and sizes. Scale bar: 100 μm.

Damaged neurons undergoing NFD are not “obvious stimuli for inflammation” as previously thought [48] since there is little evidence for glial reactions to NFD or the phagocytic removal of neurofibrillary tangles in the AD brain. In fact there is little evidence of any phagocytic activity in cases of advanced AD or DS where morphologically intact microglia or brain macrophages are difficult to discern (Figure 6). Because microglial and neuronal pathology are coincident in AD brain it is tempting to speculate that there may be a causal connection, and since there is no obvious reactive response of microglia to NFD the most likely scenario is that microglial dysfunction and death are a prelude to subsequent neuronal demise. Both microglial dystrophy and NFD are considered to be pathological events in humans where they can be detected quite early in some instances albeit without apparent neurological problems [7,89–91]. As NFD and microglial dystrophy become more widespread over time they eventually reach critical levels and become symptomatic. The progression of both processes is likely affected to varying degrees by external influences (Figure 5) and both dystrophy and NFD appear to be restricted largely to the human species and do not occur spontaneously even in aged laboratory rodents, where the main aging-related effect on both neurons and microglia is accumulation of lipofuscin.

**Figure 7:**
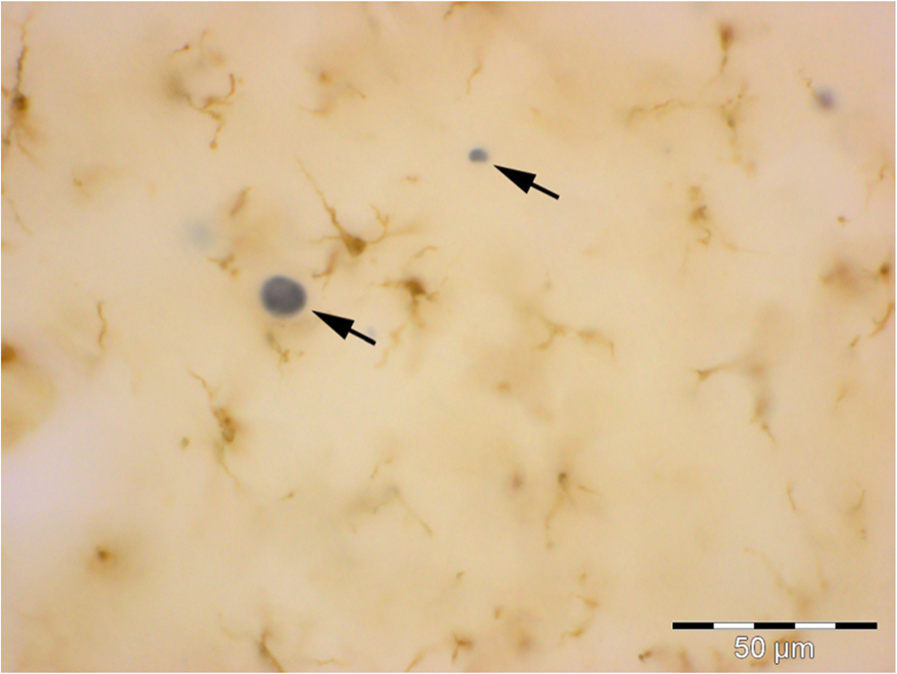
**Absence of activated microglia near neurons with Lewy bodies.** Double-label immunostaining for microglia (Iba1, brown) and α-synuclein (black) in a subject with PD, stage 5. Section of neocortex shows Lewy bodies in cortical neurons (arrows) surrounded by normal, ramified microglia.

The idea that detrimental microglial activation also contributes to the degeneration of neurons in the substantia nigra in Parkinson’s disease (PD) is firmly embedded in the literature yet much of it is speculative and extrapolated from animal and cell culture models [103–107]. In a thoughtfully delivered discussion, Croisier et al. state unequivocally that “there is no evidence that microglia initiate neurodegeneration” [108], and it would thus be fair to say that microglial involvement in PD remains a controversial and unresolved issue [109,110]. One aspect that *is* clear is the ability of microglia to phagocytize neuronal debris [108,111,112], providing a plausible explanation for presence of activated microglia/macrophages in the substantia nigra of normally aged humans [113], and certainly why microglial activation is prominent in animal models of PD where lesions and toxins are used to induce death of nigral neurons acutely [114–119]. With regard to the characteristic Lewy body pathology of PD, which is absent in most PD animal models, studies in humans have shown a lack of microglial activation in the vicinity of Lewy bodies [120,121], a finding which we have been able to corroborate (Figure 7). However, these findings contrast with those of others who do claim that activated microglia are associated with Lewy bodies and Lewy neurites and contribute neurotoxically to degeneration of nigral neurons [122,123]. It has not yet been determined whether or not presence of Lewy pathology can be correlated with appearance of microglial dystrophy.

### Microglia and amyloid-beta protein in human brain

**Figure 8:**
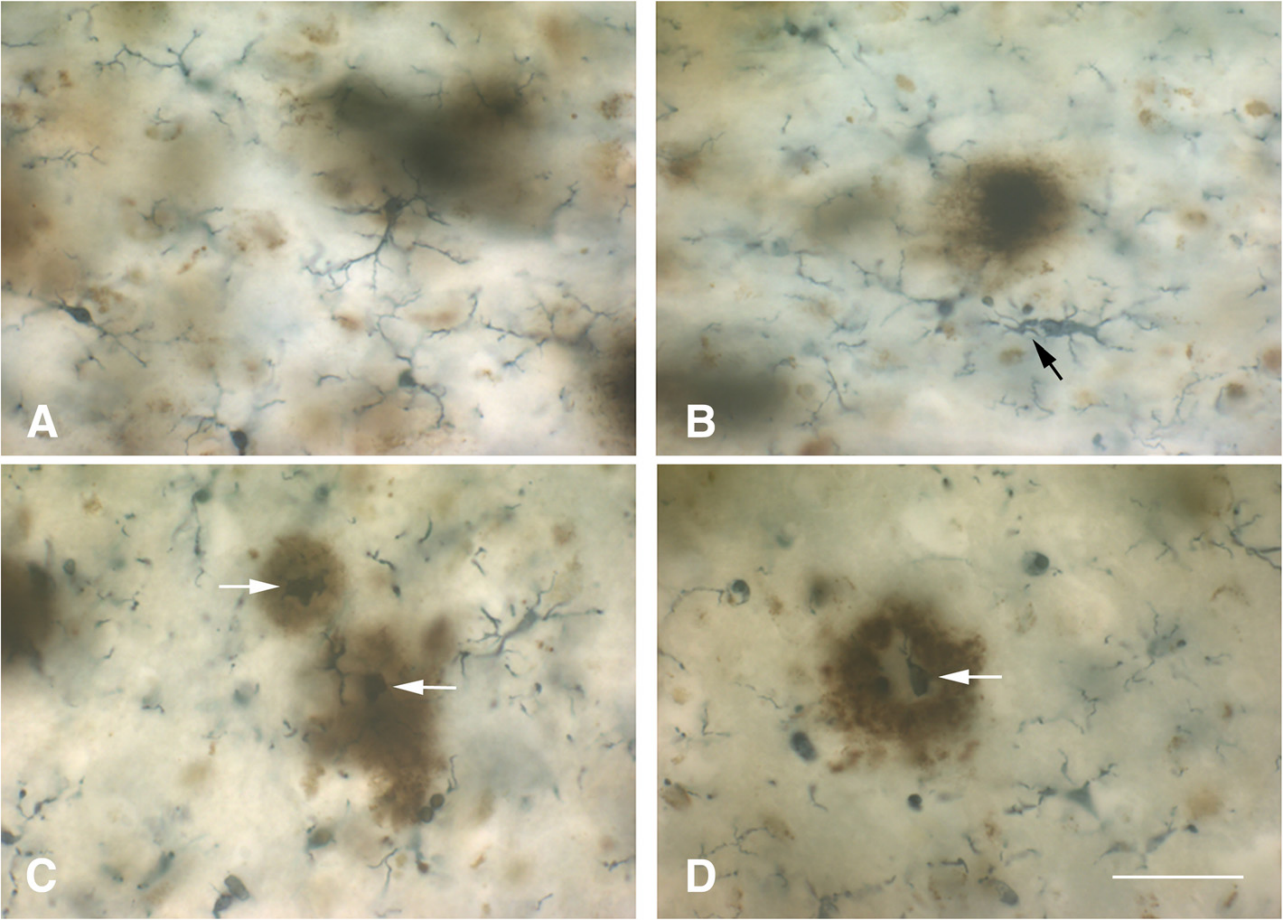
**Ramified and dystrophic microglia are associated with amyloid-beta protein deposits.** Double-label immunohistochemistry for Aβ protein (brown reaction product; 4G8 antibody) and microglia (black reaction product; Iba1 antibody) in human cerebral cortex. **A**, diffuse Aβ deposits are colocalized with ramified, non-activated microglia, **B**, as Aβ deposits become more compact microglia begin to show signs of dystrophy (arrow points to early fragmentation and spheroid formation). **C**, **D** show several advanced dense-core plaques where the dense core is comprised of dystrophic microglial fragments (white arrows). Note multiple microglial fragments in the immediate vicinty of Aβ deposits. Bar = 50 μm.

Current understanding of the relationship between microglia and amyloid-beta protein (Aβ), as well as the many questions that have arisen from it, have been discussed in detail recently [3], and in the interest of non-redundancy we focus the current discussion on one particular aspect of that relationship that has not yet received much attention, namely, that Aβ can exert detrimental effects on microglia. It is generally thought that Aβ activates microglia causing them to exert neurotoxic effects, which is a key component of the amyloid cascade theory of AD. However, and with reference to Figure 1 in [3] showing activated microglia gathered in an Aβ plaque in a transgenic mouse model, it is apparent that any activating effect of Aβ, as evidenced by the microglial hypertrophy shown in this vivid image, is limited to cells located in the immediate vicinity and along the rim of the Aβ deposit. The activating effect of Aβ is highly localized affecting only those microglia that are in direct physical contact with Aβ, whereas microglia located in the neuropil between Aβ deposits are ramified and non-activated, a pattern that is also seen in human brain [124]. The image shown in Figure 1 [3] is strongly reminiscent of a foreign body reaction, where microglia are trying to remove Aβ but are unable to do so. It may well be an example of so-called frustrated phagocytosis [87], but the consequences of this frustration may be different than thought. Traditional thinking has it that microglia activated and frustrated by contact with non-degradable amyloid plaques upregulate toxic inflammatory mediators that cause neurodegeneration [87], but this is clearly not the case in the transgenic mouse model (APPPS1) shown [3] where NFD does not occur. If NFD were present, one would expect to find it close to where activated microglia are located. A different picture also emerges if one takes a closer look at microglia in and around Aβ deposits in humans, where microglia appear as either ramified cells in diffuse (early) plaques or as dystrophic cells in neuritic (late) plaques [91] (Figure 8). In advanced plaques of the compact, dense-core, or neuritic type frustrated microglia trying to remove amyloid plaques become dystrophic and they can be seen to undergo cytorrhexis and burn out. As shown in Figure 8, dense-core plaques, also known as burned-out plaques [20], have a dense core that contains burned-out microglia and thus although “burned-out plaque” was used before anybody knew about burned-out (dystrophic) microglia, it turns out that it was a very appropriate term to use. Moreover, there are examples of previously published images showing ostensibly activated microglia in and around amyloid deposits where the cells’ morphology is rather more dystrophic than activated, e.g. [124–126], and the ultrastructure of microglia within amyloid plaques in humans is consistent with senescence revealing lipofuscin deposits, vacuolization of the cytoplasm, and swollen endoplasmic reticulum [109,124,127]. All this raises questions about whether plaque-associated microglia even become activated or how long they may remain activated before they degenerate. Head et al. [126] have shown that oxidized Aβ is found within microglia that are ostensibly activated, but are really dystrophic according to the morphology shown, and that oxidized Aβ is present in 98% of cored plaques, which supports the observations shown in Figure 8. Oxidation of Aβ may be the result of aging-related free radical damage, as it is for many other proteins and nucleic acids, or it may occur because of microglial actions as discussed in [126], but whatever the mechanisms it seems clear that oxidation of Aβ accelerates Aβ aggregation and fibrillization [128,129]. Since dystrophic microglia are associated with advanced plaques containing mostly insoluble fibrillar amyloid, the conclusion that amyloid has a detrimental or toxic effect on microglia is quite evident. Detrimental effects on microglia following exposure to Aβ peptides and to isolated human plaques have been described *in vitro* [130,131]. We therefore conclude that amyloid deposits represent an endogenous influence, in addition to the various external factors already mentioned (Figure 5), that contributes towards microglial degeneration. Aggregated Aβ in amyloid plaques together with other potentially toxic plaque components, notably iron [132–135], are likely to contribute pathologically towards dementia development by being toxic to microglia and promoting microglial degeneration which then leads to neurofibrillary degeneration.

### Causes of microglial pathology

Other than plain cellular senescence, our current understanding of mechanisms that cause or aggravate microglial dystrophy and degeneration is in its infancy. As shown in Figure 5, pure non-pathological aging as it occurs in laboratory rodents is not sufficient to trigger dystrophic changes, and other factors related to human longevity, diversity of living, and the complexity of human genetics are likely to play a role [136]. Notwithstanding this uncertainty an important clue about potential triggers of microglial degeneration was provided by observations showing that many dystrophic microglia are positive for the iron storage protein, ferritin, and that such cells are prevalent in AD brain [137,138]. Iron dyshomeostasis has long been implicated in the pathogenesis of neurodegeneration, but there is incomplete understanding of the specific mechanisms that may underlie the involvement of iron [26,139–142]. Most authors see the detrimental role of iron in its potential to cause oxidative stress more or less directly to neurons thereby contributing to neurodegeneration. In light of the foregoing discussion about microglial pathology and the importance of neuronal-glial dependency, one might rethink the role of iron and consider the possibility that the effects of iron-mediated oxidative stress may not be directed primarily or exclusively towards neurons but to microglial cells as well. Presence of free iron in the brain represents a potentially dangerous situation, which is why iron is sequestered through iron-binding proteins, such as ferritin. In situations where there is sudden entry of iron into the CNS parenchyma, for example during intracerebral hemorrhages, it is of critical importance that the iron be sequestered rapidly and effectively, and this is a critical function of microglial first responders. Experimental studies in animals have shown that when microhemorrhages occur or when there is CNS trauma, microglia not only become positive for ferritin they also become dystrophic [143,144]. The strong correlation between ferritin positivity and dystrophy, reflected in findings from both human brains as well as from experimental studies, strongly suggest that sequestration of iron is a hazardous activity that can cause damage to microglial cells. It is also an important mechanism by which microglia can protect neurons from oxidative damage, that is, microglia take the brunt of iron-mediated oxidative stress and deflect it away from neurons. Compared to neurons, microglia are relatively expendable cells since there is renewal capacity from within the CNS (mitosis) as well as from bone marrow [66,68,145–149], and one might draw the analogy to the game of chess where pawns are sacrificed to save the more valuable pieces. In terms of neurodegenerative disease pathogenesis where stroke and/or microbleeds are clearly risk factors [150–152], one can see how such vascular events can contribute to microglial degeneration and thus presumably to neurodegeneration. The situation may be aggravated further by the fact that microglial renewal capacity in aged subjects is likely to be reduced [153].

**Figure 9:**
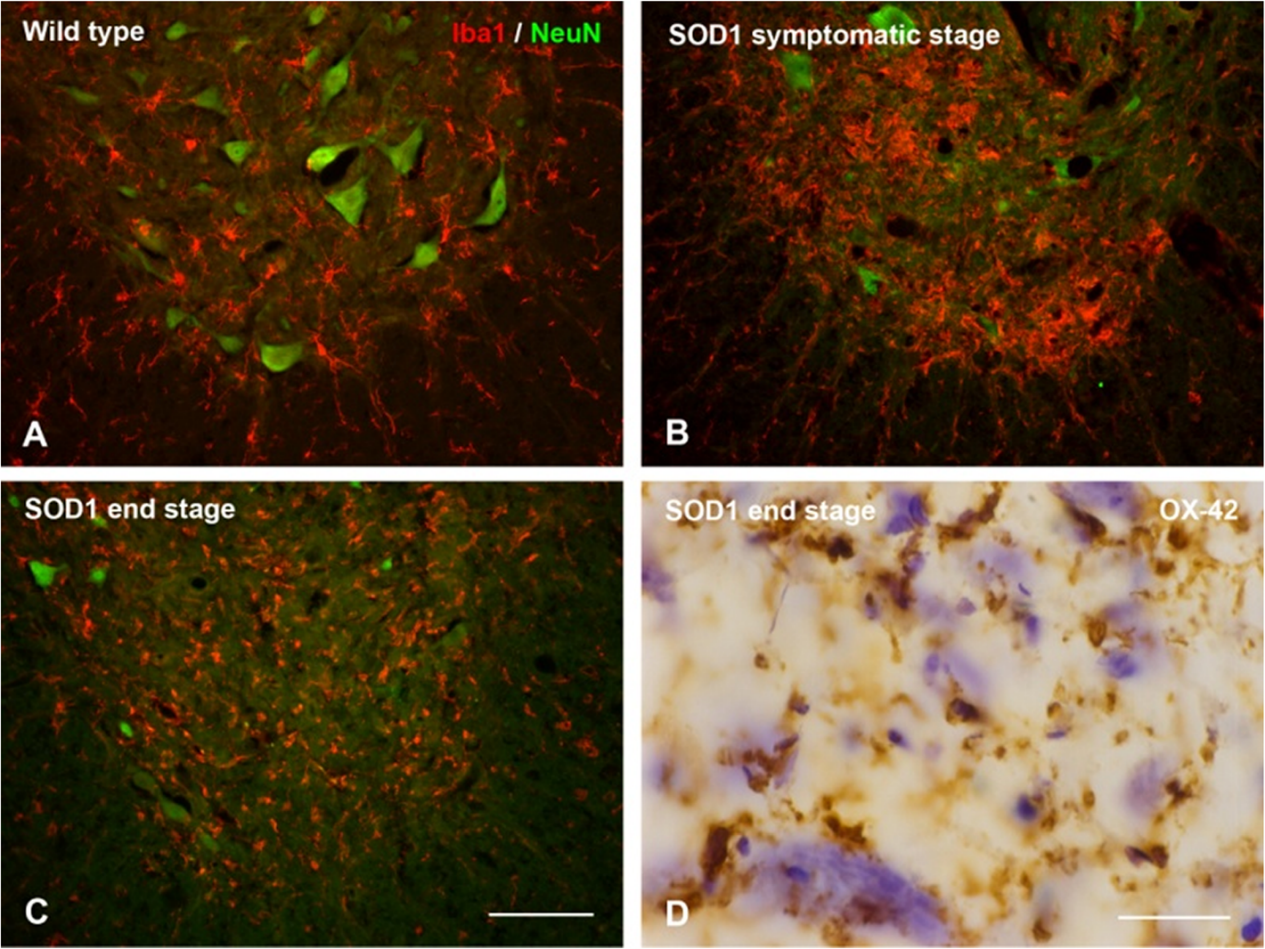
**Microglia in the spinal cord of SOD** ^**G93A**^ **rats. A-C**, double staining for microglia (Iba1) and neurons (NeuN) shows progressive loss of neurons during symptomatic and end stages of motor neuron disease. Microglial activation during symptomatic disease **(B)** progresses to microglial dystrophy at 6 months of age when animals are terminal **(C)**. Panel D shows close-up of fragmented microglia as seen with OX-42 staining (brown color) during end stage disease. A few surviving motoneurons are stained blue with cresyl violet. Scale bars: 100 μm **(A-C)**; 20 μm **(D)**.

**Figure 10:**
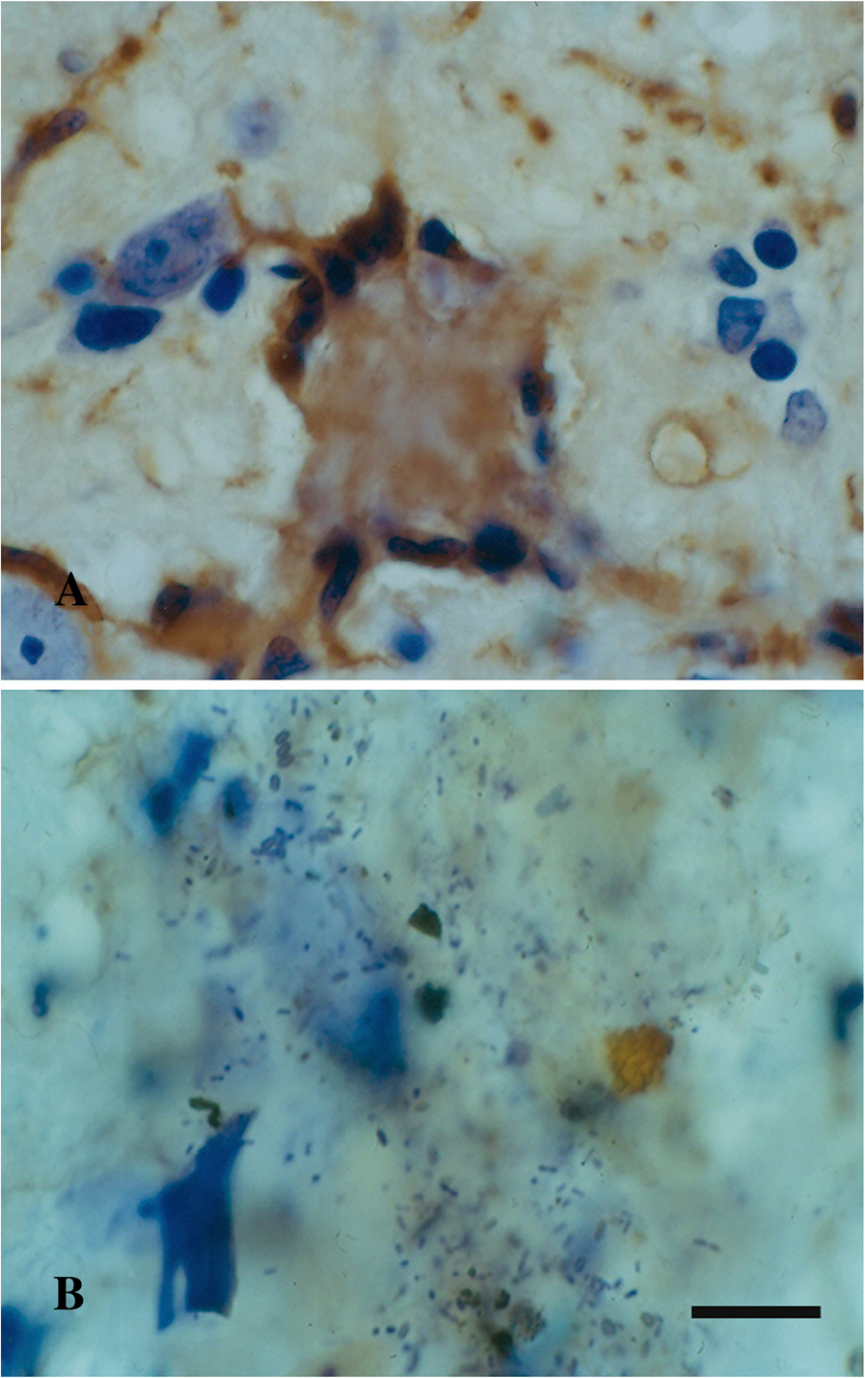
**Signs of microglial dysfunction. A**, Langhans type giant cell consisting of fused microglia in the brainstem of a SOD1^G93A^ rat, as shown by lectin histochemistry (brown color). Note the characteristic arrangement of nuclei along periphery. **B**, nidus of bacilli in another section from the same animal, possibly a reflection of impaired bactericidal activity of microglia. Cresyl violet stain. Scale bar = 20 μm.

Cytorrhexis represents the most striking example of microglial dystrophy and is indicative of an advanced, near-terminal state of cytoplasmic deterioration. Mouse models of AD do not show presence of fragmented microglia, although a recent study demonstrates other, more subtle alterations in microglial morphology amounting to cells being less ramified and possessing fewer branches and fine processes [154]. Cytoplasmic fragmentation of microglial cells has been reported in one transgenic model, the huGFAP-CCL2 transgenic mouse where microglial functions are impaired and cells fail to undergo amoeboid transformation and instead show cytoplasmic fragmentation in cortical slice preparations [155]. We have described extensive microglial cytorrhexis to occur in the SOD1^G93A^ transgenic rat, an animal model of motor neuron disease [156]. In this model, microglial fragmentation occurs late in the course of the disease after a long-lasting neuroinflammatory reaction and during the period of maximal motor neuron degeneration, thus supporting the coincidental and possibly linked development of microglial and neuronal degeneration. The fact that microglial cytorrhexis occurs after prolonged microglial activation during symptomatic motor neuron disease supports the idea that long-lasting microglial activation leads to immune exhaustion and microglial burn-out (Figure 9). However, microglial abnormalities are present at all stages of motoneuron disease in the SOD1^G93A^ rat where they are evident as cellular aggregations and fusions. Most conspicuous among the latter is the formation of Langhans-type multinucleated giant cells (MNGCs), which is a highly unusual phenomenon to observe in rat brain. In humans, MNGCs are almost always found in disease states associated with immune suppression, notably HIV encephalitis but also lymphomas and tuberculous meningitis, and are thought to occur *via* fusion of virus-infected microglia [157]. MNGCs represent a spectacular example of microglial pathology where diseased cells lose their normal contact inhibition and melt together into a non-functional syncytium. Underscoring the idea of microglial dysfunction is the fact that in SOD1^G93A^ transgenic rats MNGCs were coincident with another bizarre event in rat brain, namely the presence of bacillus bacteria (Figure 10). This observation helps consolidate the point that severe microglial disability involves compromise of both neuroprotective and immunological (bactericidal) capabilities.

**Figure 11:**
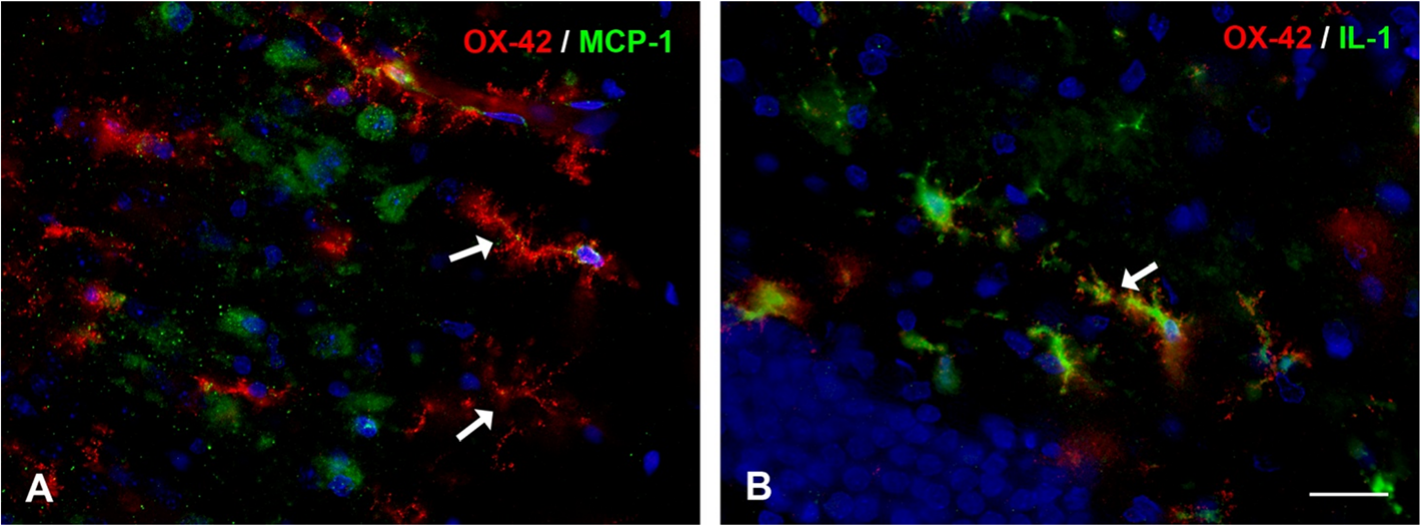
**Microglial pathology is evident in rats exposed to nerve agent.** Double immunofluorescent staining for microglia (OX-42) and cytokines (MCP-1 in A; IL-1α in B) in soman exposed rats. **Panel A** shows severe dystrophy of microglia, evident as extensive fragmentation of the cells’ cytoplasm, in the piriform cortex (arrows). MCP-1 immunoreactivity is localized in neurons. In **Panel B**, OX-42-positive microglia with fragmented processes also reveal IL-1α immunoreactivity in their cytoplasm (arrow). These cells showing features of both activation (IL-1) and degeneration (cytorrhexis) are thought to represent transitional forms; they were found in the dentate gyrus. Survival time is 12 hours; DAPI counterstain. Scale bar: 50 μm. Images provided by Erik A. Johnson, US Army Medical Research Institute of Chemical Defense (USAMRICD), Aberdeen Proving Ground, MD.

Microglial dystrophy (cytorrhexis) has also been described in another experimental model of severe neuropathology induced in rats through administration of the G-series nerve agent, soman [158]. In this model, microglia undergo extensive fragmentation often in association with high expression of proinflammatory cytokines, such as MCP-1 and IL-1 (Figure 11), and the onset of cytorrhexis as well as cytokine expression occurs rapidly within 12 hours after soman exposure. The fact that features of microglial degeneration (cytorrhexis) and microglial activation (IL-1 and MCP-1 expression) occur simultaneously in the same cells underscores the severity of soman-induced CNS injury and also offers further support in favor of microglial exhaustion, which in this case happens on a rather expedited schedule.

Overall, it appears then that microglial dystrophy can be induced experimentally in laboratory rodents under certain extreme conditions, such as genetically induced neurodegenerative disease or acute intoxication. Ultrastructural studies showing severe mitochondrial abnormalities [159] support the idea that sudden and undue oxidative stress is a key factor in the development of cytorrhexis, and thus the animal findings are consistent with the suspected influences thought to trigger microglial degeneration in humans. The idea of immune exhaustion, or microglial burn-out, is a central theme that will require further study especially since it may lend itself to remediation and therapeutic intervention.

### Significance (consequences) of microglial pathology

The study of diseased glial cells and their potential importance for neurodegenerative disease pathogenesis is in its infancy [160]. Specifically, with regard to microglia it remains a challenge to reconcile understanding of diseased, truly disabled microglia with that of activated microglia, the latter having been portrayed as overly aggressive and therefore pathogenic cells. For reasons discussed in this paper and elsewhere [88,95] it seems quite unlikely that microglial activation directly causes NFD through bystander damage, and we are inclined to believe that NFD happens as a result of gradually waning microglial neuroprotective abilities brought on in large part by immunological exhaustion of microglia, which in turn may depend on multiple factors. Microglia can be neuroprotective in a number of ways, e.g. through free iron sequestration, synapse maintenance, neurotrophic factor production, elimination of debris, and it will be interesting to determine which neuroprotective function is most crucial such that that reduction or loss could lead to NFD. However, rather than loss of a single function, it is also conceivable and perhaps even more likely that a generalized weakening in microglial capabilities below a certain minimal essential level may have to occur in order for NFD to develop. Since microglial dystrophy can be induced in animals under certain conditions as shown here, it seems possible through continued animal experimentation to uncover what kind of external factors are most effective in terms of promoting microglial exhaustion and influencing dystrophy development. These kinds of experiments may also be able to determine if indeed NFD develops as a direct result of microglial degeneration.

Multiple lines of evidence have suggested that amyloid plaques accumulate and persist because dysfunctional microglia are unable to remove them [3,56,161–163]. While this is clearly a possibility it does not explain why microglia should be phagocytically dysfunctional in the first place especially in young transgenic rodents overexpressing APP where there is no *a priori* reason for such impairment. Moreover, in human brain there is no evidence showing that microglia are attempting to remove soluble, non-fibrillized Aβ accumulating in diffuse plaques. The lack of amyloid removal may therefore not be due to impaired phagocytic ability but instead be caused by toughness and indigestibility and of the amyloid fibrils resulting in the aforementioned “frustration” which wears down the cells and contributes to their exhaustion together with other factors including naturally occurring senescence. Thus amyloid accumulation, unlike NFD development, may not be a consequence of microglial dysfunction.

Metaphorically speaking microglia are hybrids between neuroprotective glia and immunocompetent cells and their functional deterioration plays out in diminished neuroprotection as well as in decreased immunological capabilities. The latter is strikingly illustrated in Figure 10 showing presence of bacteria within the CNS parenchyma. This is a highly unusual phenomenon suggesting profound immunological impairment. Together with the coincident presence of MNGCs one thinks of immune deficiency diseases in humans, notably infection with the human immunodeficiency virus-1 (HIV-1), where MNGCs represent a pathological hallmark and microglial cells themselves are the target of the infectious agent [157,164]. It is reasonable to assume that the functionality of HIV-infected microglia is severely compromised in terms of both their neuroprotective and immunological properties, which could explain why patients with HIV/AIDS frequently suffer from the consequences of neurodegeneration, i.e. HIV-associated dementia, as well as a high incidence of opportunistic CNS infections, notably toxoplasmosis [165,166].

## Conclusions

There are aging-related morphological and phenotypic changes in rodent and human microglia that are consistent with both cell senescence and low-grade activation. However, it is all but impossible to distinguish between low-grade neuroinflammatory and senescent changes and these seemingly contradictory phenomena are in fact one and the same. Since the focus is on aging when it comes to AD, it would be wise to stop using the term neuroinflammation as this is suggestive of pathology more severe than what really exists, and therefore potentially misleading. In the context of aging it makes sense to speak simply of microglial senescence, which in humans progresses to an advanced, pathological level, called dystrophy, that can be directly associated with neurofibrillary degeneration. Making this purely semantic change from inflammation to senescence would help eliminate the rather widespread notion that microglia are aggressive immune effector cells, which is the unfortunate result of prematurely overinterpreting and extrapolating *in vitro* observations, and this has not been helpful for advancing understanding of AD pathogenesis. Microglial cells are not aggressors; they are victims of free radical damage like all cells. To help curb the ongoing dementia epidemic a major scientific challenge for the future will be to find ways of slowing or minimizing microglial senescent degeneration.
